# Oral Anticoagulant Use in Patients with Atrial Fibrillation at Low Risk of Stroke and Associated Bleeding Complications

**DOI:** 10.3390/jcm12196182

**Published:** 2023-09-25

**Authors:** Adane Teshome Kefale, Woldesellassie M. Bezabhe, Gregory M. Peterson

**Affiliations:** School of Pharmacy and Pharmacology, University of Tasmania, Hobart, TAS 7001, Australia

**Keywords:** atrial fibrillation, oral anticoagulant, stroke risk, bleeding

## Abstract

Background: The use of oral anticoagulants (OACs) in patients with atrial fibrillation (AF) and low stroke risk might cause more harm than benefit. Little attention has been given to address its prevalence and associated consequences. This study aimed to investigate the prescription rate of OACs, identify associated factors, and describe incident bleeding events in low-risk patients. Methods: We included patients with a new diagnosis of AF between 1 January 2011 and 31 December 2018 having a low risk of stroke (CHA_2_DS_2_-VASc score of 0 for males and 1 for females) from Australian general practice data (MedicineInsight). Patients were classified as OAC users if there was a recorded prescription of an OAC within 60 days of AF diagnosis, and factors associated with the prescription of an OAC were assessed using logistic regression. Recorded incident bleeding events were identified within 6 months after AF diagnosis or after OAC initiation for OAC non-users and users, respectively. The risk of bleeding was compared between the two groups by adjusting their baseline differences using propensity score matching. Results: The study included 2810 low-risk patients (62.3% male) with a mean age of 49.3 ± 10.8 years. Of the total, 705 (25.1%) patients had a record of OAC prescription within 60 days of diagnosis of AF. Older age (odds ratio [OR] 1.03; 95% confidence interval [CI] 1.03–1.04) and diagnosis periods (2015–2016 [OR 1.46; 95% CI 1.10–1.94] and 2017–2018 [OR 1.65; 95% CI 1.17–2.23] vs. 2011–2012) were associated with higher odds of OAC initiation. Female sex (OR 0.71; 95% CI 0.59–0.85), higher bleeding risk (ORBIT score; OR 0.80; 95% CI 0.68–0.94), and higher socioeconomic index for areas (SEIFA) quintiles (SEIFA quintiles; 2 [OR 0.65; 95% CI 0.48–0.88], 3 [OR 0.74; 95% CI 0.56–0.98], 4 [OR 0.70; 95% CI 0.52–0.94], 5 [OR 0.69; 95% CI 0.52–0.91] compared with quintile 1) were associated with lower odds of OAC prescription. A total of 52 (in 1.8% of patients) incident bleeds were identified, with 18 (2.6%) among OAC users. The rate of bleeding was not significantly different between users and non-users after matching. However, within OAC users, commencement of OAC was associated with an increased risk of bleeding compared to the period before OAC initiation (*p* = 0.006). Conclusions: One in four patients at low risk of stroke received an OAC within 60 days of AF diagnosis. Older age and the period following the widespread availability of direct-acting OACs were associated with an increased likelihood of OAC prescription. Positively, using OACs was not associated with an increased rate of bleeding compared to non-users.

## 1. Introduction

Atrial fibrillation (AF) has become a significant public health concern with an increasingly ageing population. Nearly one-third of stroke cases in clinical practice are associated with AF. It increases the risk of stroke up to fivefold and mortality by two times [1].

AF is an independent risk factor for thromboembolic complications [1,2]. This can, however, be significantly mitigated with the use of oral anticoagulants (OACs), through tailored initiation in patients with a high risk of stroke [1,3,4]. However, OAC therapy is associated with an increased risk of bleeding [3,4], which needs to be individually balanced against the benefit of preventing thromboembolic complications. Thus, OAC therapy is recommended in patients with a high risk of stroke, while the recommendations are against it in low-risk patients [5,6,7].

Underuse of OACs in patients with AF is well recognised [8,9,10]. Most studies have focused on assessing the prescription of OACs in patients with AF and have shown widespread underutilisation of OACs in patients with a high risk of stroke [8,11,12]. On the other hand, there is some evidence that using OACs in low-risk patients remains common in clinical practice [13,14]. In a recent study from the United States, about one in three patients with a low risk of stroke were prescribed an OAC [15], as was the case in the large quality improvement PINNACLE registry in the United States [16].

Despite recommendations of guidelines against the use of OACs in patients with a low risk of stroke [5,7], some real-world studies argue a net clinical benefit [13] and have reported a reduced risk of developing dementia [17,18]. Nonetheless, a small open-label clinical trial of 101 patients did not show any benefit of OAC therapy on either cognition or dementia over 2 years of follow-up [19]. Given the inconsistency of evidence in this patient group, the indiscriminate use of OACs potentially poses more risks than benefits, putting patients at a higher risk of bleeding. Patients with no additional non-sex risk factors for ischaemic stroke should not be prescribed an OAC, since the risk of bleeding outweighs the benefit [7]. Moreover, the use of OACs in low-risk patients might increase healthcare costs, associated with drug costs and the management of complications secondary to the therapy, particularly bleeding [20,21].

The prescription patterns of OACs in the general AF population in Australia, with an emphasis on patients with a high risk of stroke, have been published [8,22]. However, little attention has been given to describe the pattern of use in low-risk patient groups and the potential consequences. This study aimed to assess the prescription rate of OACs and identify factors associated with it in patients with AF and a low risk of stroke. Moreover, we explored the bleeding events and risk factors, using propensity score matching.

## 2. Methods

### 2.1. Data Source

The study was based on the MedicineInsight dataset, a national whole-practice data collection from consenting general practices in Australia. It included around 8.0% of all general practices. It is de-identified longitudinal data of routine practice and includes information on patient characteristics, diagnoses, pathology tests, prescribed medicines, and clinical observations extracted from the clinical information systems of general practices [23]. The dataset is representative of the Australian population in terms of age, sex, and socioeconomic status, and provides the largest comprehensive data of patients attending general practices across Australia. It has been found to be valid and complete for the most common chronic conditions [24,25]. The details of the data source are available elsewhere [25,26].

### 2.2. Study Population

The study included adults (aged ≥18 years) with a new diagnosis of AF (between 2011 and 2018) having a low risk of stroke (CHA_2_DS_2_-VASc score of 0 for males and 1 for females). Patients must have been regular attendees of the same practice site (visited the same practice site ≥ 3 times in 2 consecutive years) [27]. In addition, patients must not have had a recorded diagnosis of venous thromboembolism (VTE) within 6 months after AF.

**Figure 1:**
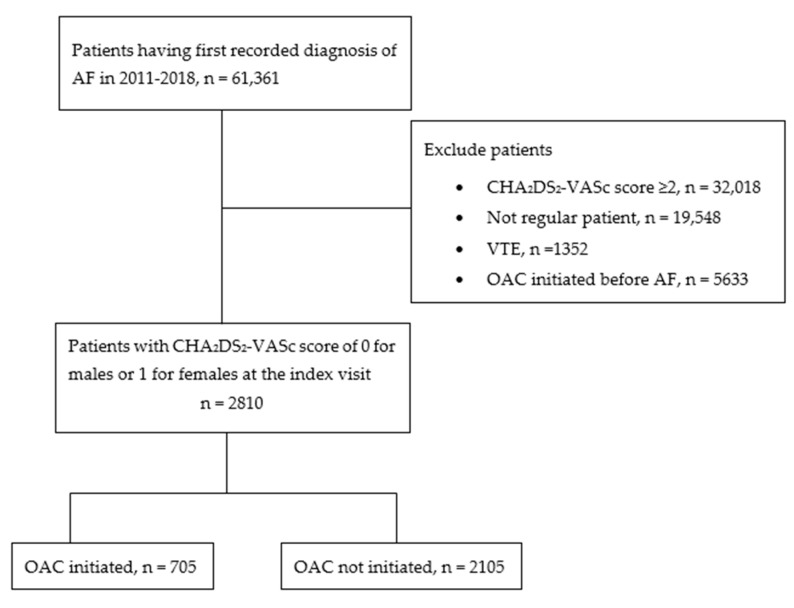
Selection of the study participants (AF: atrial fibrillation; OAC: oral anticoagulant; VTE: venous thromboembolism).

**Figure 2:**
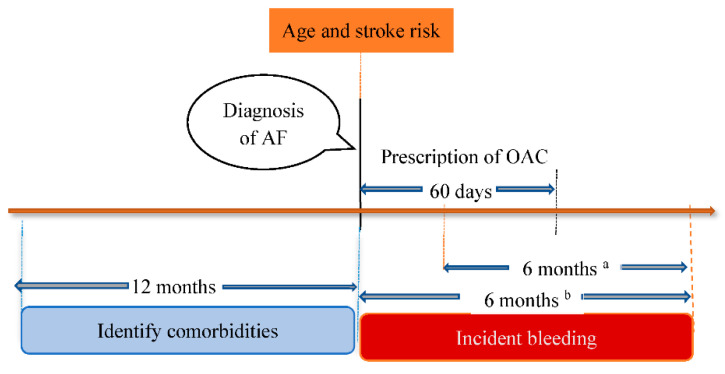
Timeline of the study (AF: atrial fibrillation; OAC: oral anticoagulant). ^a^ Follow-up for OAC users starts from the date of OAC prescription. ^b^ Follow-up for OAC non-users starts from the date of AF diagnosis.

The date on which the diagnosis of AF was recorded for the first time in the study period was considered an index visit, which was a cohort entry date for the study. The details of the patients’ selection process and the timeline of the study are presented in Figure 1 and Figure 2, respectively.

Bleeding events were assessed by following patients for 6 months after OAC initiation, and any records of bleeds were extracted. Other covariates were collected during the 12-month period preceding the index visit (Figure 2).

### 2.3. Study Variables

The age and CHA_2_DS_2_-VASc score (Congestive heart failure [1 point], Hypertension [1 point], Age ≥ 75 years [2 points], Diabetes mellitus [1], Stroke/transient ischaemic attack [2 points], Vascular disease [1 point], Age 65–74 years [1 point], and Sex category [female, 1 point]) [28] were calculated at the first record of being diagnosed with AF (the index visit). In the primary analysis, OAC use was defined based on the prescription of the OAC within 60 days from the index visit. Sensitivity analysis was conducted within 180 and 365 days of the index visit by recalculating the stroke risk.

The risk of bleeding was calculated at the index visit using the Outcomes Registry for Better Informed Treatment (ORBIT) score (Older age ≥ 75 years [1 point], Reduced haemoglobin [2 points], Bleeding history [2 points], Reduced renal function [estimated glomerular filtration rate (eGFR) < 60 mL/min, 1 point], and Treatment with an antiplatelet [1 point]). Patients were categorised as either having a low (ORBIT score 0–2), medium (score 3), or high (score ≥ 4) risk of bleeding [29]. The most recent values (relative to the index visit) of blood haemoglobin and eGFR were extracted from the records. Anaemia was considered when the haemoglobin level was below 130 g/L in males and 120 g/L in females.

Comorbidities were identified using coded and uncoded terms provided by MedicineInsight [23] and according to an algorithm [18]. Incident bleeding events were extracted from the records as condition flags and text searches. Terms for text searching and an algorithm were developed to identify bleeding events (Supplementary Table S1). Incident bleeding events were assessed within 6 months of the index visit and OAC initiation date for non-users and users of OACs, respectively. Moreover, we compared the rate of bleeding in OAC users for 6 months pre- and post-OAC initiation.

The rurality was determined from the address of the general practice (postcode) using the Australian Statistical Geography Standard (ASGS) of the Australian Bureau of Statistics (ABS) [30]. The ASGS classifies areas into five rurality categories based on relative access to services, measured using the Accessibility and Remoteness Index of Australia (ARIA+). The ARIA+ score estimates the road distance of a point to the nearest urban centres and localities. The five categories are major cities (ARIA+ 0–0.20), inner regional (ARIA+ 0.21–2.40), outer regional (ARIA+ 2.41–5.92), remote (ARIA+ 5.93–10.53), and very remote (ARIA+ > 10.53) [30]. The last two were combined into one group in this analysis. The socioeconomic status of participants was estimated using the ABS Socio-Economic Indexes for Areas (SEIFA) decile based on the general practice’s postcode [31]. The SEIFA ranks areas from 1 (the most disadvantaged) to 10 (the most advantaged). This analysis classified the SEIFA into quintiles (five groups) derived from the deciles.

### 2.4. Statistical Analysis

Proportions and means with standard deviations were used to summarise categorical and continuous variables, respectively. The proportion of patients with a low risk of stroke who were prescribed an OAC within 60 days of their index visit was calculated. A chi-square test was used to describe the differences in patients with and without an OAC prescription for categorical variables, whereas an unpaired *t*-test was used for continuous parametric variables. A McNemar test was used to compare the rate of bleeding in OAC users during pre- and post-OAC initiation.

Propensity score matching was used to balance the differences in baseline characteristics between users and non-users of OACs. The rate of bleeding events between those prescribed and not prescribed an OAC was compared after matching. We performed a 1:1 matching between the two groups using their propensity scores. The scores were calculated using the logit of the propensity score at a matching calliper of 0.25 [32]. Once the propensity score was calculated, each OAC user was matched with non-users, using an optimal matching method. The balance of baseline patients’ characteristics was compared before and after matching using an absolute standardised mean difference at a cut-off value >0.10 for a significant imbalance between the two groups.

Multivariable logistic regression was modelled to control confounding in describing the difference between users and non-users of OACs. The model was adjusted for sex, age, SEIFA quintiles, comorbidities (listed in Table 1), indexing periods, and ORBIT score. Age and ORBIT score were treated as continuous variables in the logistic model. Variables with a *p*-value < 0.1 were included in the final model, with backward elimination of variables with a higher *p*-value. Multicollinearity between independent variables was checked using a chi-square test. We ran two different models, pre- and post-collinearity tests. Variables were excluded in the second model when the *p*-value was <0.05 during the chi-square test. We kept age and sex in the model regardless of *p*-values, as these variables are known to be key factors in determining OAC use. Dementia was not included in the model due to small numbers that did not fulfil the assumption for regression (>20% of cells with expected counts less than 5). Moreover, renal function, Aboriginal and Torres Strait Islander (ATSI) status, and abnormal haemoglobin were excluded initially from the model because of the large proportion of missing values. Data were analysed using SAS version 9.4 (SAS Institute Inc., Cary, NC, USA), and statistical significance was considered at a two-sided *p*-value < 0.05 at a 95% confidence interval (CI).

## 3. Results

### 3.1. Sociodemographic Characteristics of Study Participants

A total of 2810 AF patients (mean age 49.3 ± 10.8 years, 62.3% male) who met the eligibility criteria were included in the final analysis. As we included patients with a non-sex CHA_2_DS_2_-VASc score of 0, all patients had no recorded diagnosis of hypertension, diabetes mellitus, stroke, or vascular disease, and all were aged below 65 years. The most common comorbidities were depression (24.6%) and arthritis (22.8%). The bleeding risk, according to ORBIT, at the index visit was low in 99.3% of patients (Table 1).

### 3.2. Prescription Patterns of OAC in Low-Risk Patients

Of 2810 patients, 705 (25.1%) patients were prescribed an OAC within 60 days of diagnosis of AF. There were differences across sex, age, indexing periods, socioeconomic status, and common comorbidities between users and non-users of OAC (Table 1). Rivaroxaban was the most common OAC prescribed (267; 37.9%), followed by apixaban (207; 29.4%), warfarin (174; 24.7%), and dabigatran (57; 8.1%).

In the sensitivity analysis, the number of patients prescribed an OAC increased slightly to 836 (29.8%) and 931 (33.1%) within 180 and 365 days of diagnosis of AF, respectively.

### 3.3. Determinants of OAC Prescription in Low-Risk Patients

We fitted multivariable logistic regression models to identify predictors of OAC prescription, utilising two scenarios to account for multicollinearity between covariates. In the second model, we excluded chronic liver disease (CLD), depression, and chronic obstructive pulmonary disease (COPD), as these were strongly associated with either age or sex (*p* < 0.001). Both models found consistent results regarding predictors. Accordingly, increasing age by 1 year and the indexing period between 2015–2018 were associated with higher odds of OAC use, while higher SEIFA quintiles were negatively associated with OAC prescription. Moreover, an increment of ORBIT score by 1 was associated with a 20% less likelihood of receiving an OAC (Table 2).

### 3.4. Incident Bleeding Events

During the 6 months of follow-up, 52 (1.8%) patients with at least one bleeding event were identified. Gastrointestinal and urogenital bleedings were the most common types of incident bleeds recorded (Table 3). The proportions of patients with bleeds were not significantly different between OAC users (2.6%) and OAC non-users (1.6%; *p* = 0.14). Of the total bleeding events among users, 8, 5, 3, and 2 were identified with warfarin, dabigatran, rivaroxaban, and apixaban, respectively. After matching to account for potential confounders, the rate of incident bleeding was not significantly different between OAC users and non-users (*p* = 0.47). The characteristics of patients after matching are detailed in Supplementary Table S2.

In addition, the risk of bleeding was compared among OAC users by examining the 6-month periods pre-and post-OAC commencement. Four cases of bleeding were recorded in the 6 months preceding OAC initiation, while 18 bleeds in different individuals were recorded within 6 months after OAC initiation. Using the McNemar test, OAC initiation was strongly associated with an increased risk of bleeding (OR 4.50; 95% CI 1.48–18.28, *p* = 0.006).

## 4. Discussion

This was a real-world cohort of 2810 patients with AF having a low risk of stroke, from general practices enrolled in MedicineInsight from States and Territories in Australia. The study found that over one-quarter of patients had received at least one prescription of an OAC within 60 days of AF diagnosis. This finding is consistent with a study conducted in the United States using the PINNACLE registry of 6730 patients with a CHA_2_DS_2_-VASc score of 0. That study reported that 23.6% of patients were prescribed an OAC [14]. In another large PINNACLE registry of 12,348 patients with a low risk of stroke [16], the prevalence of OAC prescribing corroborated our and the earlier findings. This is in contrast to a study conducted in the Netherlands, which suggested that OACs were prescribed to 58% of the patients with a CHA_2_DS_2_-VASc score of 0 [33]. Unlike our study, however, it investigated OAC prescription within 1 year of AF diagnosis, which broadened the prescription period. Clinical practice guidelines recommended against the use of OAC in patients with a low risk of stroke [5,7]. A multicentre retrospective cohort study of 59,076 patients from Denmark, Norway, Scotland, and Sweden found net clinical benefits of OACs in low-risk patients [13]. However, the findings might have been skewed, as patients with moderate risk were also included.

As was the case in higher-risk patients [9,22,34], the use of OACs in low-risk patients was higher after the introduction of direct-acting oral anticoagulants (DOACs) into clinical practice. This might be secondary to clinicians’ assumptions of the relative safety of DOACs compared to warfarin. However, evidence shows that DOACs do not differ in major bleeding rates compared to warfarin, despite their lower risk of intracranial haemorrhage. Moreover, DOACs are reported to increase the risk of gastrointestinal bleeding [3]. Positively, increasing age was associated with the prescription of an OAC, as it would be in moderate- to higher-risk patients [15]. This could be due to the increasing prevalence of coronary heart disease (CHD) with age, as shown by our data, where increasing age was strongly associated with the presence of CHD (*p* < 0.001).

The risk of bleeding is one of the major factors influencing the decision to prescribe OAC therapy. It is the most common reason reported not to initiate an OAC, including for patients with high stroke risk [35]. Physicians usually give higher weight to the risk of bleeding than the risk of stroke in deciding on an OAC prescription [35]. We found that OAC prescription was 20% less likely with each 1-point increase in the ORBIT score. Female patients with a low risk of stroke were 30% less likely to receive an OAC than males. Several other reports have shown females were less likely to be prescribed an OAC [36,37,38,39], regardless of stroke risk, despite tending to have an increased risk of stroke compared to males [37]. Similarly, patients with better socioeconomic status had a 25–35% lower likelihood of receiving an OAC. One explanation could be that patients with higher economic conditions are usually living in a metropolitan area, where general medical practitioners might have better exposure to cardiologists, health information, and the latest treatment guidelines.

We also assessed the rate of incident bleeding and compared the two groups. Overall, the incidence of bleeding was 1.8% and slightly higher in OAC users (2.6%), which is in line with a study from Sweden (2.23%) [40], although the follow-up was for 12 months and only major bleeding was assessed in the latter study. However, in our study, the difference in bleeding rate was not statistically significant between users and non-users of OAC. Nonetheless, the McNemar association test among the users during the pre- and post-OAC initiation period showed that there was a 4.5-fold increase in incident bleeding following OAC initiation. Of the total bleeding events among users, the largest proportion was in patients prescribed warfarin, consistent with previous studies [3,21]. A small number of cases, which may not be powerful enough to estimate the true difference in the rate of bleeding, limited us from conducting further analysis. The data also did not provide reliable information to assess the severity of bleeding.

The study has limitations to be considered. The dataset includes prescription data from general practice, which cannot necessarily indicate that patients took an OAC, even if prescribed. As the study employed general practice data not linked with hospital data, the incidence of bleeding might be underreported and solely depends on the quality of recordkeeping by general practices. It was also not possible to grade the severity of bleeding and accurately identify specific types of bleeding. Although the first few months after OAC initiation are prone to adverse events, such as bleeding, the cumulative incidence increases with time on treatment [41]. The 6-month follow-up for bleeding might have been too short to estimate the true rate of bleeding in practice. Future study is recommended using longer follow-up periods and linked hospital data to assess stroke and bleeding outcomes.

## 5. Conclusions

One in four patients with AF were initiated on OAC therapy without an evidence-based indication. Older age and the prescription periods from 2015 to 2018 were positively associated with OAC initiation, whereas female patients and those who were living in higher-socioeconomic-status areas had lower odds of initiating OACs. Considering the limitations with the data used, the rate of incident bleeding did not show a significant increase associated with OAC use. However, in a paired comparison within the users before and after OAC initiation, there was an increase in the proportion of bleeding following OAC prescription.

## Figures and Tables

**Table 1:** Baseline characteristics of patients included in the study.

Characteristic	All, n (%)	OAC Users (n = 705)	Non-Users (n = 2105)	*p*-Value
Sex, male	1750 (62.3)	477 (67.7)	1273 (60.5)	<0.001
Age, mean (SD) years	49.3 (10.8)	51.9 (9.2)	48.4 (11.1)	0.022
Diagnosis period				<0.001
2011–2012	408 (14.5)	75 (10.6)	333 (15.8)	
2013–2014	613 (21.8)	128 (18.2)	485 (23.0)	
2015–2016	788 (28.1)	214 (30.4)	574 (27.3)	
2017–2018	1001 (35.6)	288 (40.8)	713 (33.9)	
Indigenous status	n = 2192	n = 558	n = 1634	0.746
ATSI	51 (2.3)	14 (2.5)	37 (2.3)	
Non-ATSI	2141 (97.7)	544 (97.5)	1597 (97.7)	
Missing	618 (22.0)	147 (20.8)	471 (22.4)	
SEIFA quintiles	n = 2790	n = 699	n = 2091	0.020
1	384 (13.8)	122 (17.4)	262 (12.5)	
2	498 (17.9)	112 (16.0)	386 (18.5)	
3	644 (23.1)	161 (23.0)	483 (23.1)	
4	552 (19.8)	132 (18.9)	420 (20.1)	
5	712 (25.5)	172 (24.6)	540 (25.8)	
States and territories				0.396
ACT	67 (2.4)	16 (2.3)	51 (2.4)	
NSW	1084 (38.6)	291 (37.7)	793 (41.3)	
NT	36 (1.3)	11 (1.6)	25 (1.2)	
QLD	551 (19.6)	145 (20.6)	406 (19.3)	
SA	87 (3.1)	20 (2.8)	67 (3.2)	
TAS	190 (6.8)	47 (6.7)	143 (6.8)	
VIC	495 (17.6)	112 (15.9)	383 18.2)	
WA	300 (10.7)	63 (8.9)	237 (11.3)	
Rurality	n = 2797	n = 701	n = 2096	0.108
Major cities	1684 (60.2)	419 (59.8)	1265 (60.4)	
Inner regional	794 (28.4)	188 (26.8)	606 (28.9)	
Outer regional	257 (9.2)	80 (11.4)	177 (8.4)	
Remote/very remote	62 (2.2)	14 (2.0)	48 (2.3)	
Comorbidities				
Anxiety	571 (20.3)	121 (17.2)	450 (21.4)	0.017
Arthritis	641 (22.8)	188 (26.7)	453 (21.5)	0.006
Asthma	454 (16.2)	114 (16.2)	340 (16.2)	1.000
COPD	98 (3.5)	34 (4.8)	64 (3.0)	0.032
Depression	692 (24.6)	149 (21.1)	543 (25.8)	0.013
Dementia	4 (0.1)	1 (0.1)	3 (0.1)	1.000
CLD	27 (1.0)	3 (0.4)	24 (1.1)	0.118
Cancer	535 (19.0)	145 (20.6)	390 (18.5)	0.430
Anaemia Missing	60 (3.3) 991 (35.3%)	22 (4.8) 250 (35.5)	38 (2.8) 741 (35.2)	0.047
CHD	155 (5.5)	50 (7.1)	105 (5.0)	0.036
eGFR in mL/min	n = 1931	n = 502	n = 1429	0.305
≥60	1823 (94.4)	468 (93.2)	1355 (94.8)	
45–59	84 (4.4)	27 (5.4)	57 (4.0)	
30–44	15 (0.8)	3 (0.6)	12 (0.8)	
<30	9 (0.5)	4 (0.8)	5 (0.4)	
Missing	879 (31.3)	203 (28.8)	676 (32.1)	
ORBIT Score, mean (SD)	0.41 (0.60)	0.37 (0.61)	0.43 (0.59)	0.021
ORBIT risk category				0.914
Low	2789 (99.3)	699 (99.2)	2090 (99.3)	
Medium	18 (0.6)	5 (0.7)	13 (0.6)	
High	3 (0.1)	1 (0.1)	2 (0.1)	

ATSI: Aboriginal and Torres Strait Islander; CHD: coronary heart disease; CLD: chronic liver disease; COPD: chronic obstructive pulmonary disease; eGFR: estimated glomerular filtration rate; OAC: oral anticoagulant; ORBIT: Outcomes registry for better informed treatment; SD: standard deviation; SEIFA: socioeconomic index for areas. States and territories: ACT: Australian Capital Territory; NSW: New South Wales; NT: Northern Territory; QLD: Queensland; SA: South Australia; TAS: Tasmania; VIC: Victoria; WA: Western Australia.

**Table 2:** Factors associated with prescription of OAC in patients at low risk of stroke.

Variables	Model 1		Model 2	
	AOR (95% CI)	*p*-Value	AOR (95% CI)	*p*-Value
Sex				
Male	Ref	Ref	Ref	Ref
Female	0.72 (0.60–0.86)	<0.001	0.71 (0.59–0.85)	<0.001
Age	1.03 (1.02–1.04)	<0.001	1.03 (1.03–1.04)	<0.001
Diagnosis period				
2011–12	Ref	Ref	Ref	Ref
2013–14	1.17 (0.85–1.62)	0.339	1.15 (0.83–1.58)	0.402
2015–16	1.49 (1.12–1.98)	0.006	1.46 (1.10–1.94)	0.001
2017–18	1.65 (1.20–2.29)	0.002	1.61 (1.17–2.23)	0.004
SEIFA quintile				
1	Ref	Ref	Ref	Ref
2	0.65 (0.48–0.89)	0.007	0.65 (0.48–0.88)	0.006
3	0.75 (0.56–1.00)	0.050	0.74 (0.56–0.98)	0.034
4	0.70 (0.52–0.94)	0.019	0.70 (0.52–0.94)	0.018
5	0.70 (0.53–0.93)	0.013	0.69 (0.52–0.91)	0.009
ORBIT score	0.80 (0.68–0.93)	0.005	0.80 (0.68–0.94)	0.005
Comorbidities				
Depression	0.82 (0.66–1.01)	0.067		
CLD	0.26 (0.08–0.90)	0.032		
COPD	1.46 (0.94–2.26)	0.096		

AOR: adjusted odds ratio; CI: confidence interval; CLD: chronic liver disease; COPD: chronic obstructive pulmonary disease; SEIFA: socioeconomic index for areas.

**Table 3:** Types of bleeding events identified within 6 months of OAC initiation.

Types of Bleeding	OAC Users, n = 705	OAC Non-Users, n = 2105
Gastrointestinal bleeding	3	14
Urogenital bleeding	7	10
Haematoma	3	4
Subconjunctival haemorrhage	1	3
Intracranial haemorrhage	1	
Nasal bleeding	2	1
Anaemia secondary to blood loss	1	
Haemoptysis		2
Total	18	34

OAC: oral anticoagulant.

## Data Availability

Data sharing is restricted by the owner of the dataset; thus, data other than those included in the manuscript will not be publicly available.

## Supplementary Materials

The following supporting information can be downloaded at: https://www.mdpi.com/article/10.3390/jcm12196182/s1, Table S1: Terms used to search bleeding events; Table S2: Characteristics of patients before and after matching.
