# Supplementary material for: Oral Anticoagulant Use in Patients with Atrial Fibrillation at Low Risk of Stroke and Associated Bleeding Complications
Source: J Clin Med. 2023 Sep 25;12(19):6182. doi: 10.3390/jcm12196182 (PMC10573191; doi:10.3390/jcm12196182)
Supplement: Supplementary file 1 [file jcm-12-06182-s001.zip › jcm-2598475-supplementary.pdf]

Supplementary tables

Table 1: Terms used to search bleeding events

Outcome	Searching terms
Bleeding	Abnormal bleeding; abscess formation in R leg haematoma; acute dysfunctional uterine bleeding; acute left epistaxis; Anaemia -blood loss; anaemia, blood loss; ante partum haemorrhage; bike accident-subarachnoid bleed; bleeding – gastrointestinal tract; bleeding – post menopausal; bleeding – vaginal; bleeding from anus; bleeding from lesion on left arm; bleeding GIT; bleeding gums; bleeding haemorrhoids; bleeding in early pregnancy; bleeding PR; bleeding PV; bleeding spot; bleeding via rectum; bleeding, vaginal; blood in stool; blood in stools; blood stained diarrhoea; bloods RV; breakthrough bleeding; breast lump PR bleeding; BTB (break through bleeding); chest wall haematoma; cough – blood; cough having blood stained phlegm/x-ray chest; cough mild haemoptisis; diarrhoea with blood; drainage of thrombosed haemorrhoid; DUB (dysfunctional uterine bleeding); dysfunctional uterine bleeding; ear -bleeding; epistaxis; Excision, PR bleed; extensive bleed into lower R leg; faeces – occult blood; gastrosym, bleed; GIT bleeding; Haemarthrosis; Haemarthrosis -Knee; Haemarthrosis of Knee; Haematemesis; haematemesis episode; haematoma; haematoma – undiagnosed; haematoma - incision of; Haematoma – right groin; haematoma – subungual drainage of; Haematoma medial mid r lower leg; haematospermia; haematuria; haematuria - microscopic; haematuria – undiagnosed; haematuria, frank; haematuria – macroscopic; haematuria, macroscopic; haematuria, microscopic; haematuria-no recurrence; haematuria, painless gross; hemoperitoneum; haemoptysis; haemorrhagic gastritis; haemorrhoidal bleeding; haemorrhoids / bleeding; haemothorax; hysterectomy for dysfunctional bleeding; INR / rectal bleeding; intermenstrual bleeding; knee Haemarthrosis; left conjunctival haemorrhage; left Haemarthrosis – Knee; left haematoma; left subconjunctival haemorrhage; left temporal intraparenchymal haematoma; macroscopic haematuria; melaena; metrorrhagia; microscopic haematuria; mild epistaxis; mild, acute haematospermia; mild, acute rectal bleeding; moderate, acute epistaxis; N bloods; nosebleed; nose bleed; occult blood in stool; P/V bleeding; PAP, bleeding PR, atrophic vagina; pelvic pain and PV bleed; perianal haematoma; peri anal haematoma; peri-anal haematoma; post coital bleeding; post coital bleeding with large cervical erosion; post coital bleeding; postcoital bleeding; post menopausal bleeding; postmenopausal vaginal bleeding; post operative bleeding; post partum bleeding; PR bleed; PR bleed – FIA; PR bleeding; prolonged bleeding; prolonged PV bleeding; PV bleed; PV bleeding; PV bleeding in early pregnancy; PV bleeding post IUD; rectal bleeding and vaginal bleeding and ulcer buttock; R eye posterior bleeding; 2 episodes of rectal bleeding; rectal bleeding; rectal bleeding undiagnosed; rectal blood loss; rectal mucus and blood; reported haematuria; retinal haemorrhage; right conjunctival haemorrhage; right ear – bleeding; right epistaxis; right haematoma; right haematoma, periorbital; right subconjunctival haemorrhage; sinusitis, right thrombosed ext haemorrhoid; skins, bloods RV; small external

	haemorrhoid- with old thrombosis; sputum with blood; stools – blood; subconjunctival haemorrhage; subconjunctival haemorrhage (left) subconjunctival bleed; subcutaneous haematoma of suprapubic region and sharp of peni; subdural haematoma; subdural haematoma -acute; subdural haematoma – chronic; subdural haemorrhage; subungual haematoma; subungual haematoma drainage; thinning blood; thrombosed external haemorrhoid; thrombosed ext haemorrhoid; thrombosed ext. haemorrhoid; thrombosed haemorrhoid; upper GI bleed; vaginal bleeding – postmenopausal; vaginal bleeding – postpartum; vomiting – blood; wound bleeding

Table 2: Characteristics of patients before and after matching

Characteristics	Before matching			Propensity score matched		
	OAC users (n =705)	Non-users (n =2105)	Standardised differences*	OAC users (n =699)	Non-users (n =699)	Standardised differences*
Age, mean (SD)	51.9 (9.2)	48.4 (11.1)	0.34	51.9 (SD 9.1)	51.4 (SD 9.6)	0.045
ORBIT score, mean (SD)	0.37 (0.61)	0.43 (0.59)	0.12	0.37 (0.61)	0.38 (0.62)	0.023
Sex, male	477 (67.7)	1273 (60.5)	0.15	473 (67.7)	488 (69.8)	0.045
Anxiety	121 (17.2)	450 (21.4)	0.11	119 (17.0)	113 (16.2)	0.022
Arthritis	188 (26.7)	453 (21.5)	0.12	187 (26.8)	182 (26.0)	0.017
Asthma	114 (16.2)	340 (16.2)	0.005	114 (16.3)	112 (16.0)	0.0078
Depression	149 (21.1)	543 (25.8)	0.11	148 (21.2)	142 (20.3)	0.020
Cancer	145 (20.6)	390 (18.5)	0.052	144 (20.6)	142 (20.3)	0.0072
CHD	50 (7.1)	105 (5.0)	0.089	50 (7.2)	45 (6.4)	0.030
CLD	3 (0.4)	24 (1.1)	0.081	3 (0.4)	2 (0.3)	0.016
COPD	34 (4.8)	64 (3.0)	0.092	34 (4.9)	32 (4.6)	0.015

CHD: coronary heart disease; CLD: chronic liver disease; COPD: chronic obstructive pulmonary disease; OAC: oral anticoagulant

*All standardised differences were measured as absolute mean differences
